# Exosome: emerging biomarker in breast cancer

**DOI:** 10.18632/oncotarget.16684

**Published:** 2017-03-29

**Authors:** Yunlu Jia, Yongxia Chen, Qinchuan Wang, Ushani Jayasinghe, Xiao Luo, Qun Wei, Ji Wang, Hanchu Xiong, Cong Chen, Bin Xu, Wenxian Hu, Linbo Wang, Wenhe Zhao, Jichun Zhou

**Affiliations:** ^1^ Department of Surgical Oncology, Sir Run Run Shaw Hospital, Zhejiang University, Hangzhou, Zhejiang, China; ^2^ Biomedical Research Center and Key Laboratory of Biotherapy of Zhejiang Province, Hangzhou, Zhejiang, China; ^3^ Brown University, Providence, Rhode Island, USA; ^4^ Department of Radiology, Second Affiliated Hospital, Zhejiang University, Hangzhou, Zhejiang, China

**Keywords:** breast cancer, exosome, metastasis, drug resistance, cancer therapy

## Abstract

Exosomes are nano-sized membrane vesicles released by a variety of cell types, and are thought to play important roles in intercellular communications. In breast cancer, through horizontal transfer of various bioactive molecules, such as proteins and mRNAs, exosomes are emerging as local and systemic cell-to-cell mediators of oncogenic information and play an important role on cancer progression. This review outlines the current knowledge and concepts concerning the exosomes involvement in breast cancer pathogenesis (including tumor initiation, invasion and metastasis, angiogenesis, immune system modulation and tumor microenvironment) and cancer therapy resistance. Moreover, the potential use of exosomes as promising diagnostic and therapeutic biomarkers in breast cancer are also discussed.

## INTRODUCTION

Breast cancer is one of the most common malignancies in women [1]. According to report of the American Cancer Society, breast cancer alone take over 29% all new cancer diagnoses in women in 2016, and it is expected to be leading cause of cancer-related death in women aged from 20 to 59 years [2]. Past decades have witnessed various cutting-edge diagnostic and prognostic methods and the improvement of novel targeted therapies due to major advances in our deeper comprehension of breast cancer biology. Currently, available treatments efficacy of breast cancer is still limited by drug toxicity and resistance and lack of dependable predictive and prognostic biomarkers. Thus, accelerating progress against breast cancer requires both the development of novel biomarkers and therapeutic targets and further understanding of the potential molecular mechanisms.

Exosomes are 40-100nm diameter membrane vesicles of endocytic origin, which are secreted by various kinds of cells and contain a broad repertoire of cargoes, including nucleic acids (ex., DNA, mRNA, miRNA, long and short noncoding RNA), proteins (ex., cytoskeletal proteins, Transmembrane proteins, and heat shock proteins), and enzymes (GAPDH, ATPase, pgk1) [3]. Generally, contents of exosomes can reflect the nature and status of original cells. The upgrading of specific proteins and nucleic acids implies a degree of specific cellular sorting into exosomes [4]. On the other end of spectrum, exosomes have the ability of modulating cellular activities in recipient cells by transferring genetic information [5, 6, 7].

Recently, research on the role of exosomes involved tumorigenesis and cancer progression has grown exponentially, including immune suppression, angiogenesis, cell migration and invasion [6–8]. As exosomes are capable of transferring specific proteins and nucleic acids to recipient cells in the tumor microenvironment or at specific distant sites, cancers have used exosomes as a tool by which cancer cells can transfer malignant phenotype to normal cells, and establish a fertile local and distant microenvironment to help cancer cell growth [9]. Contents as miRNA and proteins found in tumor-derived exosomes isolated from patients’ bodily fluids play an important role in cancer development and progression [10] [11]. These features render exosomes as potential biomarkers for liquid biopsy, and utilizing exosomal profiling in the absence of tissue hold pronounced promise for disease early diagnose and therapy efficacy monitoring.

Increasingly, exosomes are studied for their potential roles as both indicators of breast cancer and a prospective new treatment approach. Exosomes can play important roles in different stages of development in breast cancer. Through horizontal transfer of genetic information between breast cancer cells, exosomes are supposed to exhibit pleiotropic biological functions, including stimulating tumor angiogenesis, reorganizing the stroma to establish the tumor microenvironment, as well as promoting tumor growth and drug resistance. This comprehensive review highlights our understanding of the contribution of the exosomes during breast cancer development and evolution. We will also discuss key concepts on their possible clinical applications, including their use as prognosis biomarkers and novel therapeutic targets, such as drug delivery system and exosome-based vaccine.

## BIOGENESIS AND CHARACTERISTICS OF EXOSOMES

**Figure 1:**
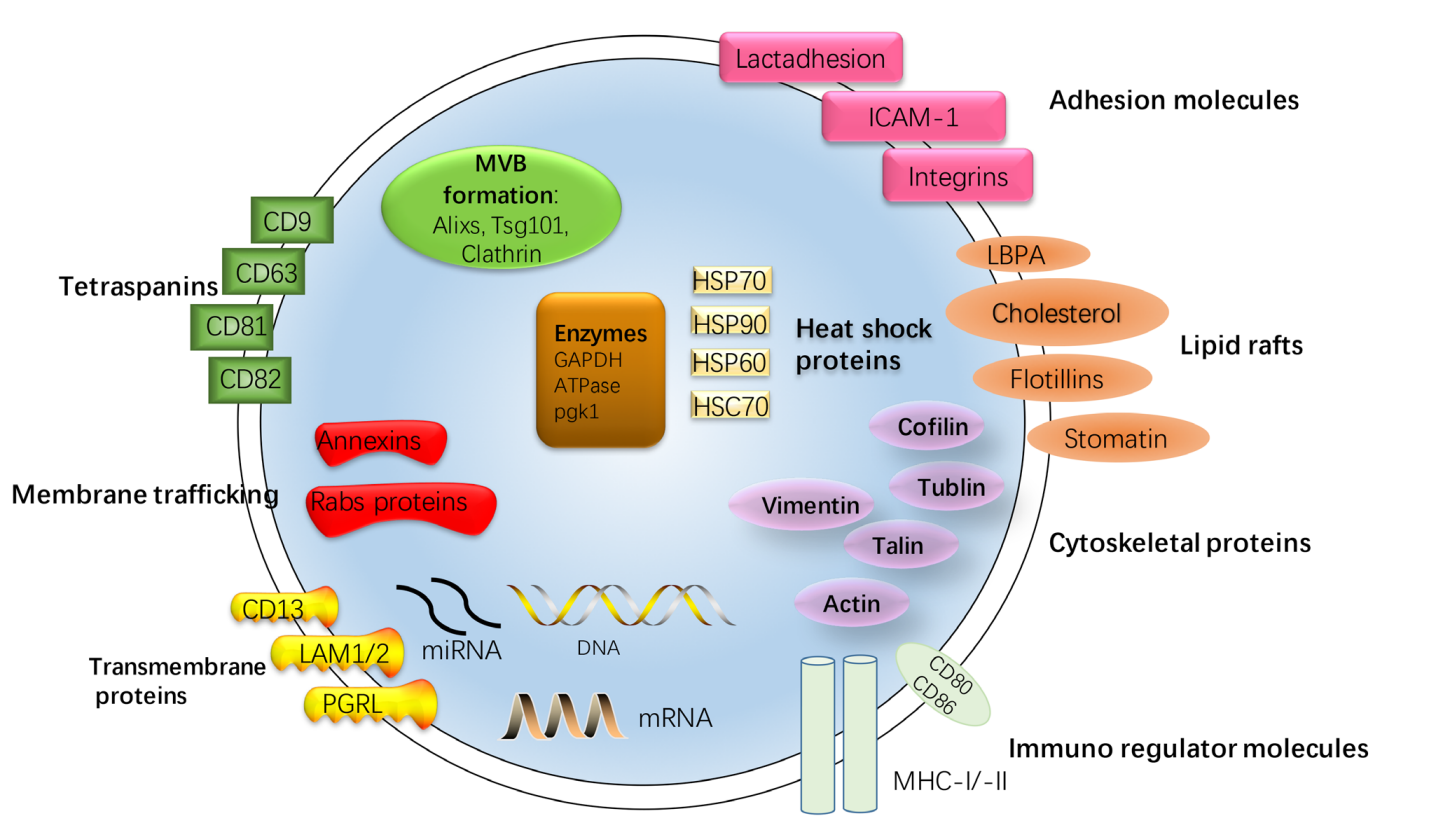
Structure and contents of exosomes Exosomes are small membrane bound vesicles sharing similar topology to the plasma membrane. The lipid bilayer membrane structures of exosomes carry contains typical transmembrane proteins and receptors, such as the transmembrane proteins PGRL, LAMP1/2, CD13, membrane trafficking proteins as annexins and RABs proteins, adhesion molecules as ICAM-1, Lactadhesion and intergrin, the lipid raft associated protein as Flotillins, LBPA, Cholesterol, stomatin and tetraspanins, most characteristically CD63 and CD81. Furthermore, the exosomal membranes surface is assembled with Immuno regulator molecules, such as MHC-I and -II, CD80 and CD86. Within the exosomal lumen several proteins stabilizing and conserving the ‘informative’ exosomal cargo can be found: Cytoskeleton proteins, HSPs, most typically Hsp70, cytoskeletal proteins (Vimentin, Cofilin, Tublin, Talin, Actin), metabolic enzymes (GAPDH, ATPase, pgk1) and protein involving in MVB formation as Alixs, Tsg101, Clathrin. The contents of exosomes can be transferred from the original cell to their target cells in microenvironment, consisting of RNA species (mRNAs, miRNAs and other types of short RNAs), and a vast array of different proteins depending on their host cell.

Exosomes are membrane vesicles of endocytic origin, which shaped from inward budding of membrane of multi-vesicular bodies (MVB) and released from the cell into the extracellular environment with the plasma membrane. Most prokaryotic and eukaryotic cells can release exosomes, including stroma cell, reticulocytes, epithelial cells, and tumor cells, which have been isolated from serum, urine, bile, and breast milk [3–5] [7] [12]. According to proteomic analyses, exosomes have been verified to contain a selective enrichment of a discrete set of cellular protein associated with cell surface receptors, cytosolic signaling proteins, metabolic enzymes, antigen presentation, and major histocompatibility complex (MHC), heat shock protein (HPSs, as HSP70, HSP90, HSP60, HSP70), and tetraspanins [13, 14]. Although previously considered as cellular waste products, recent studies have showed that exosomes carry amount of functional molecules, provide shelter to the transported molecules and act as intercellular correspondents by shuttling molecules between cells [12] [15] [16]. Here, the structure and contents of exosomes is demonstrated in Figure 1.

**Figure 2:**
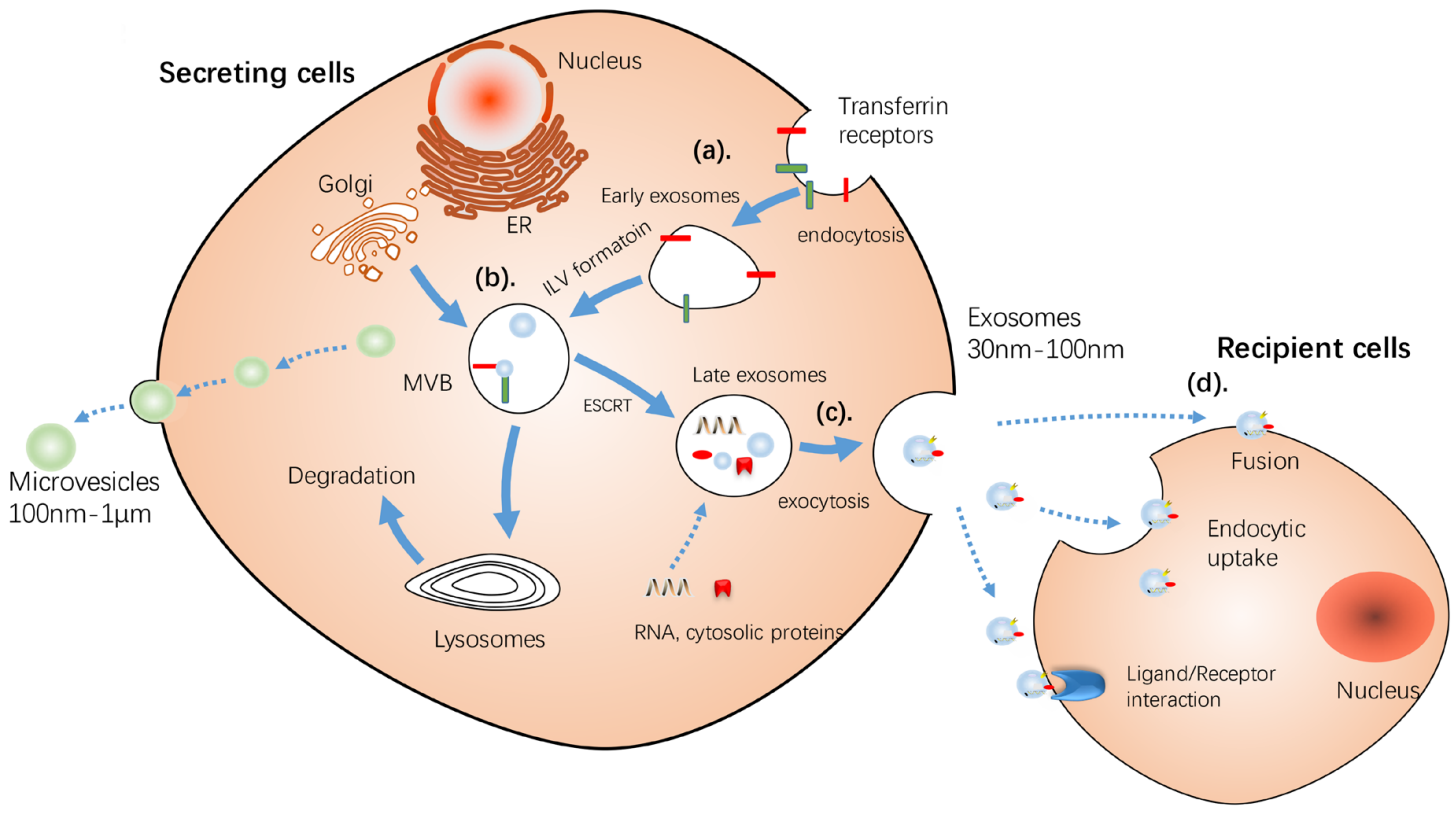
Schematic illustration of Release of MVs and exosomes MVs bud directly from the plasma membrane, whereas exosomes are repressed by small vesicles of different sizes that are formed as the ILV by budding into early endosomes and multivesicular body and are released by fusion of multivesicular body fuse with lysosomes. **a**. By endocytosis of membrane segments, the initial endosome arises, containing receptors and transmembrane proteins of the plasma membrane. **b**. Instead of lysosomal degradation, the matured late endosome transforms by inward budding of tiny vesicles into a multivesicular body. Furthermore, the exosomal cargo as proteins and miRNA, is selectively loaded into the vesicles. **c**. MVs bud directly from the plasma membrane, whereas the exosomes are released into the extracellular space by fusion of the multivesicular body with the plasma membrane **d**. Cell-secreted exosomes can travel through biological fluids (e.g. serum, lymph) and be internalized by recipient cells. Three different sorting complexes have been identified so far: the ESCRT pathway with its associated proteins ALIX and TSG101, the ceramide-dependent sorting complex and a tetraspanin-mediated sorting pathway including CD63, CD81 and TSPAN8. ESCRT: endosomal sorting complex required for transport machinery. MT, mitochondria; MVB, multivesicular bodies.

There are a large number of mobile membrane-limited vesicles called extracellular vesicles (EVs) in the extracellular environment, which can be further subcategorized based on their size, biogenesis and release mechanism, and content into exosomes, microvesicles (MVs), and apoptotic bodies [3] [17]. Exosomes and MVs have a distinct biogenesis, while the most dramatic difference between exosomes and MVs is how they are formed [18]. Exosomes are formed from inward budding of an endosome resulting in a multivesicular body (MVB), with the plasma membrane, which is secreted by succeeding fusion of the MVB. On the other hand, MVs are released directly by budding from the cellular plasma membrane (detailed difference between exosome and MVs are summarized in Table 1) [19–22]. Moreover, most of discussed studies claimed that their studies were based on exosomes. Henceforth, exosomes and microvesicles are collectively referred to as exosomes unless indicated. (The release process of MVs and exosomes is showed Figure 2)

**Table 1:** Difference between exosomes and MVs

	Exosomes	MVs
Size and Shape	30-100nm; a characteristic round or cup-shape	100nm – 1000nm; composed of a round-shaped, heterogeneous population
Formation	Budding to the inside of late endosomes	budding towards the outside from the plasma membrane
Components	Heat shock proteins (HSP70, HSP90, HSP60, HSC70) Tetraspanins (CD9, CD63, CD81, CD82) Membrane trafficking (Annexins, Rabs proteins) Adhesion molecules, Cytoskeletal proteins, Immuno regulator molecules, Enzymes, miRNA, mRNA and DNA	Phosphatidylserine, Integrins, Selectins, CD40 ligand

Several studies has indicated the potential mechanisms by which exosomes are internalized and released. Accumulating evidence has indicated that impose stress on cells, growth factors and increases in intracellular calcium can induce the release of exosomes [23, 24]. Exosomes also exert a feedback mechanism regulating the release of exosomes from normal mammary, it means that exosomes released from breast cancer cells and normal human mammary epithelial cells are regulated by exosomes derived from their own cells [25]. Other investigations have revealed that that exosomes are internalized *via* phagocytosis or lipid raft domains [26] [27]. In breast tumor cells, detachment of cells is a intense stimulation for the secretion of exosomes [28]. Exosomes have also been expected to contributed to cell-cell communication by activating them directly by surface expressed ligands or by conveying signal molecules between cells. To be specific, exosomes interact with target cells *via* receptors, endocytosis, fusion with plasma membrane, or the release of their cargo [29, 30]. Cancer cell derived exosomes usually carry molecular signs and effectors of the disease, such as mutant onco-proteins, oncogenic transcripts, microRNA, and DNA sequences. When taken up by recipient non-malignant cells, such exosomes contribute to horizontal cellular transformation and phenotypic reprograming, traverse the tumor microenvironment, and finally result in the cell malignant transformation [14, 31]. What is more, as the content of exosomes is closely connected to the original cells which the exosomes are derived, exosomes are increasingly considered as novel diagnostic or prognostic biomarkers [32]. Recently, growing number of studies have also revealed the important role of exosomes as both indicators of cancer development and a prospective new treatment approach in breast cancer.

## TUMOR TRANSFORMATION

During initial malignant transformation, exosomes generated by breast cancer cells contain a variety of proteins and RNA species can be transfected between cancer cells as well as cancer and normal cells, conferring a transformed-like phenotype to normal mammary epithelial cells. Although the exact underlying mechanisms remain to be elucidated, many research findings have revealed that exosomes could alter the transcriptomes of target cells and contribute to oncogenic transformation and tumor formation [33] [34]. For example, exosomes secreted by breast caner cell (MDA-MB-231) were capable of transforming normal human mammary epithelial cells (MCF10A cells) into cancer cells [35]. In cell culture and mice models, these cancer exosomes contained miRNAs (miR-10b and miR-21) altered the transcriptome of recipient cells, with the RNA-induced silencing complex (RISC)-loading complex proteins (RLC), and process pre-miRNAs Dicer, TAR RNA-binding protein 2 (TRBP) and Argonaute-2 (AGO2) into mature miRNAs. In addition to exosomes acting locally to promote tumor formation and proliferation, they can also impact cells at distant sites through their ability to affect cell migration and invasion capacity.

## HALLMARKS OF TUMOR GROWTH, INVASION AND MIGRATION

**Figure 3:**
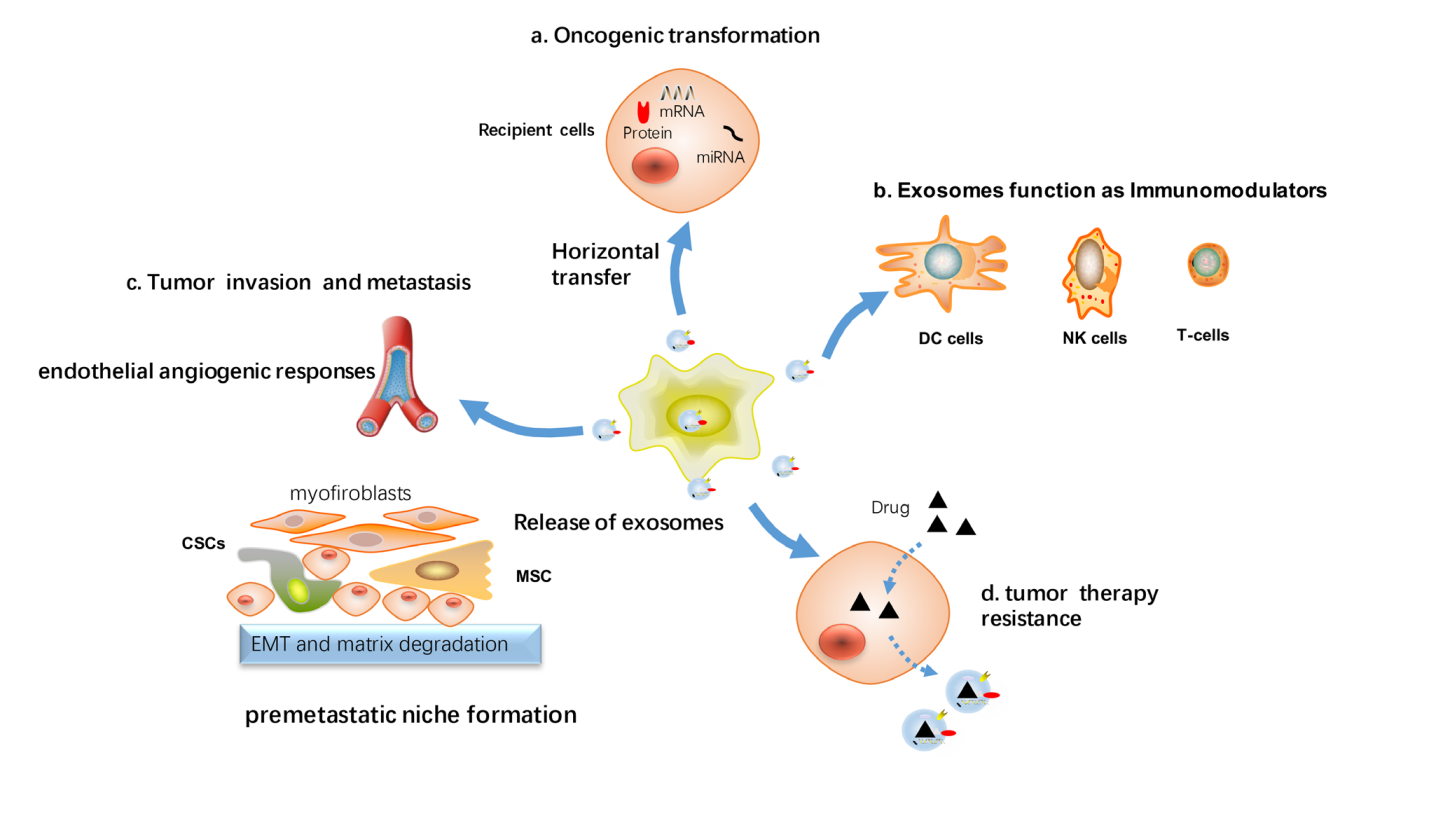
Cellular processes affected by exosomes-mediated signaling in breast cancer Tumor cells and stromal cells exchange exosomes carrying proteins and nucleic acids that can affect the function of recipient cells. **a**. *Via* horizontal transfer of protein or RNA, exosomes can contribute to the spread of the transformed phenotype from tumor cells to surrounding normal cells. **b**. Inhibition of the immune response against tumor cells by inhibiting the proliferative response of immune cells (DC cells, NK cells and T-cells). **c**. Exosomes influence tumor invasion and metastasis, as stimulate endothelial angiogenic responses, epithelial to mesenchymal transition (EMT), activate cancer stem cell and preparation of a premetastatic niche at the distant location. **d**. Induction of cancer therapy resistance including chemoresistance, radiation resistance, endocrine and target therapy resistance.

In breast cancer, in addition to taking part in initial malignant transformation, exosomes can transfer signaling molecules to cancer cells within the tumor microenvironment, and help tumor cells evade immune response, promote tumor invasion and metastasis, remodel the tumor microenvironment, and stimulate angiogenesis (Figure 3).

Metastasis requires cell manipulate local environment to optimize invasion and growth, including loss of adhesion, increased migration and invasion [36–38]. Adhesion is extremely important not only in various pathological conditions but also in cancer biology. In breast cancer cells, cellular detachment is related with significant release of exosomes, and then exosomes concentrate on the cell surfaces and mediate adhesion to extracellular matrix proteins [28] [39]. Fetuin-A, a glycoprotein from fetal bovine serum, has been proved to provide a significant clue regarding cellular adhesion [40]. *Via* recruiting exosomes, Fetuin-A can mediate cancer cells adhesion [41], and these exosomes also contribute to the preparation of the metastatic niches and regulate cell growth and motility [42] [43]. Taken together, these currently available data show that exosomes isolated from breast cancer cells can stimulate cell migration and induce migration proportional to the metastatic potential of the cell [44].

Plenty of research have shown that miRNAs secreted from exosomes improve the invasive and migration capacity of several breast cancer cell lines. Kruger *et al*. investigated exosomes derived from MDA-MB-231 encompassed a higher level of matrix metalloproteinase proteins, while the exosomes derived from MCF-7 contained more nucleic acid, transfer proteins, and protein binding. In addition, the Gene Ontology analysis revealed that several of profiled miRNAs are related with tumor progression and metastasis, and a bundle of miRNAs (including miR-301a, miR-130a, miR-34a, miR-106b, miR-328 etc.) had different expression levels between MDA-Exo and MCF-Exo [45]. Exosome-mediated miR-10b secretion suppressed the protein level of its target genes such as HOXD 10 and KLF4 and induced invasion ability in breast cancer cell [46]. Characteristically expressed and secreted by metastatic breast cancer cells, miR-105 regulated cell migration through targeting ZO-1 [47]. Breast cancer cells secreted miR-122 promoted cell metastasis by adjusting the metabolic environment to a premetastatic niche. By inhibiting glucose uptake by premetastatic niche cells, exosomal miR-122 reprogramed systemic energy metabolism and enhanced cancer development [48]. In addition, tumor-secreted exosomal miR-9, upregulated in various breast cancer cell lines and known as a pro-metastatic miRNA, can be taken up by normal fibroblasts and switch to cancer-associated fibroblasts phenotype, thus contributing to tumor growth [49]. Bone metastasis is a frequent event in breast cancer, and transfer of exosomal miRNAs is functional mediators of tumor-stromal communications in breast cancer bone metastasis. Authors identified four exosomes-secreted miRNAs (miR-127, -197, -222, and -223) that inhibited CXCL12 expression in co-cultured MDA-MB-231 and T47D to suppress cell proliferation and stimulate cell cycle quiescence [50]. In breast cancer cells, exosomal miR-23b promoted dormancy and decreased CD44 surface abundance, a characteristic marker of breast cancer stem cells (CSCs). Exosomal transfer of miR-23b from the bone marrow and its suppression of MARCKS caused breast cancer cell quiescence in metastatic niches [51].

Triple-negative breast cancers (TNBC) are defined as lack of expression of progesterone receptor (PR) and oestrogen receptor (ER) and amplification of human epidermal growth factor receptor 2 (HER2) gene. TNBC derived exosomes could confer phenotypic traits to secondary cells *via* cell-to-cell communication. Study has revealed TNBC cell line Hs578Ts(i)_8_-exosomes significantly improved the proliferation, migration and invasion abilities of the recipient cell lines, and stimulated sensitivity to *anoikis* [52]. The preoperative blood serum levels of miRNA from patients with breast cancer revealed association between the level of exosomal miR-373 to triple negative and more aggressive breast cancer. Exogenous overexpression of miR-373 in MCF-7 cells caused downregulated protein expression of RE, and suppression of apoptosis induced by camptothecin [53]. Exosomes derived from noncancerous cells can also influence cancer progression. Wnt signaling pathway can directly stimulate exosomes secretion and participate in the transportation of exosomal molecules in breast cancer progression. Fibroblast-secreted exosomes promoted breast cancer cell invasion and metastasis by stimulate epithelial-mesenchymal transition (EMT) and matrix degradation *via* Wnt-planar cell polarity signaling [54]. Macrophages promoted invasiveness of breast cancer cells *via* deliver invasion-potentiating miR-223, *via* regulation of the Mef2c-β-catenin pathway [55]. Major exosomal miRNAs involved in breast tumor progress is exhibited in Table 2, including their target genes and mainly physiological function.

**Table 2:** Major miRNAs involved in breast tumor progress

miRNA/Protein	target genes	physiological function
miR-130a	TGB-β/Smad signaling	tumorigenesis
miR-328	CD44	reduce cell adhesion and enhance cell migration
miR-301a		a negative prognostic maker
miR-34a	p53	
miR-106b	BRMS1; RB	an early process of tumor metastasis
miR-10b	HOXD10; KLF4	transfer to non-maligant HMLE cells and promote cell invasion
miR-105	ZO-1	cell migration and metastasis
miR-127, -197, -222, and -223	CXCL12	suppress cell proliferation and elicit cell cycle quiescence
miR-23b		promote cell dormancy in a metastatic niche
miR-373		downregulate ER expression and inhibition of apoptosis
miR-122		inhibit glucose uptake in a premetastatic niche and promote metastasis
miR-9		transfer cancer-associated fibroblasts phenotype to normal fibroblasts
miR-223	Mef2c–β-catenin pathway	promote invasiveness of breast cancer
miR-16	VEGF	suppress angiogenesis
miR-210		key factors for the tumor angiogenesis and brain metastasis
miR-451, miR-326, miR-100, miR-222, and miR-30a		drug resistance
miR-221/222		enhance tamoxifen resistance
miR-155, miR-21 and miR-1246,	the shelterin component TERF1	cancer diagnose and predict a poor prognosis
miR-215, miR-299, and miR-411		lower expression in untreated patients with metastatic breast cancer
miR-155, miR-19a, miR-181b, and miR-24		an early marker for breast cancer risk
miR-101, miR-939, miR-373		breast tumor subtype and stage

## EXOSOMES INFLUENCE ON TUMOR GROWTH UNDER HYPOXIC ENVIRONMENT

**Figure 4:**
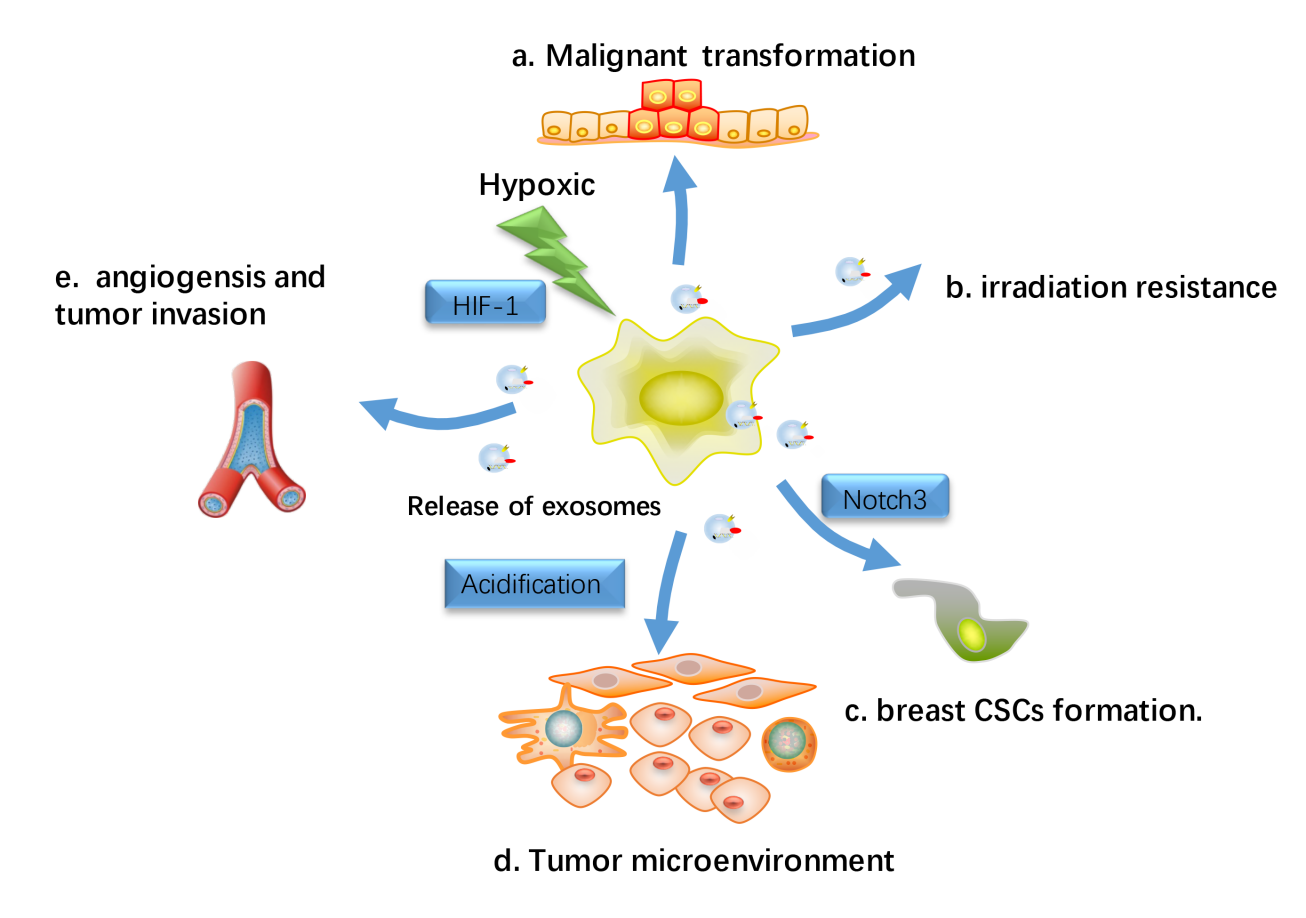
Hypoxic effects on exosomes-Influenced metastasis Hypoxia has been regard as a major trigger for the release of exosomes release in breast cancer cell, and the hypoxia-induced release of exosomes from cancer cells is hypothesized to result in the malignant transformation and subsequent proliferation and migration of normal recipient cells. **a**. Transfer miRNA or protein to recipient cells and lead to malignant transformation in normal cells. **b**. Hypoxic tumor microenvironments render cancer cells more resistant to irradiation. **c**. Hypoxic induced exosomes induce the stem cell regulatory gene Notch3 in breast CSCs **d**. Hypoxia resulted in acidification of the tumor microenvironment, which may have a profound influence on exosomes release and uptake. **e**. facilitate angiogenesis and tumor metastasis.

Hypoxic, an often transient phenomenon presents at microscopic sites within tumors microenvironment, which is linked to angiogenesis, tumor aggressiveness, treatment resistance and poor outcomes in a range of different cancers. With a unique ability, cancer cells can survive and grow under hypoxic environments [56]. In breast cancer cells, hypoxia condition is regarded as a major trigger for exosomes secretion [57, 58]. Under hypoxic conditions, the effects of exosomes on tumor angiogenesis and growth are even more noticeable [59–61] (Figure 4). It has been reported that hypoxia-induced release of exosomes from cancer cells may lead to neoplastic transformation, malignant cell growth and invasion [62–64]. Hypoxia was also favorable for exosomes trafficking due to tumor microenvironment acidification, as exosomes release and uptake have been indicated to be facilitated at lower pH [65]. Park et al. revealed that hypoxic or re-oxygenated epidermal carcinoma cells secreted exosomes with the potential to regulate cell microenvironment and improve angiogenesis and metastasis [66]. This result was recapitulated in breast cancer. In breast cancer, hypoxia-mediated triggering of HIF-1α (Hypoxia inducible factors) enhanced exosomes secretion and lead to aggressive cell phenotype [67]. Breast cancer-derived exosomes produced under hypoxic conditions can also promote inflammatory crosstalk within the tumor niche to promote growth, which is decreased by activation of nuclear receptors within the cancer cell [68]. Besides, tumor cells made cancer cells significantly reduce the effectiveness of irradiation under hypoxic tumor microenvironments. It has been reported hypoxic regions in breast cancers is more resistant to radiation and chemotherapy [69].

## EXOSOMES CONTRIBUTE TO TUMOR PROGRESSION *VIA* MODULATING STEM CELLS

Mesenchymal stem cells (MSCs) have the potential for self-renewal and act as precursors for tumor stroma including myofibroblasts, which are key cells of tumor stroma and play an important role in the tumor angiogenesis. [70] [71]. Karnoub *et al* have suggested an interaction between breast cancer progression and MSCs [72]. Tumor-derived exosomes can contribute to breast cancer development by converting adipose tissue-MSCs into tumor-associated myofibroblasts, and exosome treatment can increase the expression of tumor-stimulating molecules (SDF-1, VEGF, CCL5 and TGFβ) [73]. Ruizhu Lin *et al* have verified MSC secreted exosomes significantly promoted MCF-7 migration *via* triggering Wnt signaling pathway [74]. MCF-7-derived exosomes also modulated breast cancer stem cells (CSCs) formation. Notably, MCF-7 derived exosomes significantly induced the stem cell regulatory gene NOTCH3 in breast CSCs under hypoxic microenvironment [68]. MCF-7-derived exosomes up-regulated the activation marker CD44, the CSCs growth factor IL6, as well as ApoE. In another study, 4T1 mouse breast derived exosomes functioned as pro-tumorigenic factors by promoting the cell growth and suppression apoptosis of CD133 + tumor stem cells [75].

## EXOSOMES ACT AS STIMULATING FACTORS FOR TUMOR ANGIOGENESIS

Exosomes are important mediators in promoting cell angiogenesis. Previously study has suggested that breast cancer cells derived exosomes converted MSMs into myofibroblasts *via* a SMAD-mediated pathway [76]. Myofibroblasts, which are key cells of tumor stroma and play an important role in the tumor angiogenesis. Thus, Exosomes from breast cancer cells tumor exosome-induced recruitment of fibroblasts could support tumor angiogenesis. Breast cancer cell-derived exosomal miR-16 downregulated VEGF expression in cancer cells and suppressed angiogenesis, which could be consider as an effective anti-angiogenetic agent for anti-tumor therapy [77]. The correlation between circulating miR-210 and tumor presence and lymph node metastases in breast cancer patients has been well established. Neutral sphyngomyelinase 2 (nSMase2) affected exosomal miRNA secretion and promoted cancer cell metastasis through the induction of angiogenesis in the tumor microenvironment. Specifically, exosomal angiogenic miRNAs from metastatic breast cancer cells, such as miR-210, promoted their metastatic initiation efficiency *via* enhancing endothelial cells migration and capillary formation [78]. Exosomal miR-210 might be one of the key factors for tumor angiogenesis and brain metastasis, and high expression of miR-210 predicted poor survival in breast cancer patients with brain metastatic [79, 80].

## INTERACTION BETWEEN EXOSOMES AND TUMOR MICROENVIRONMENT IN CANCER PROGRESSION

Tumor cells can create microenvironments (premetastatic niche) suitable for cancer distance metastasis [81]. Tumor cell-derived exosomes act as a vehicle for the horizontal transfer of exosomal miRNAs and protein between epithelial cells, stromal cells, macrophages, and fibroblasts and tumor cells, manipulating the tumor microenvironments to establish premetastatic niche which is more permissive for cancer invasion and migration [82] [83]. Moreover, authors proposed that tumor-derived exosomes might contribute to metastatic niche form to promote tumor metastasis [84] [85].

The cell factors secreted to the tumor microenvironment from preadipocytes and adipocytes are important components of the cancer stem cell niche and exert their oncogenic effects *in vivo* [86]. Cancer-derived extracellular miR-122 convert the metabolic environment to a premetastatic niche, by suppressing cells glucose uptake and reprograming systemic energy metabolism [48].

More recently, by injecting mice with exosomes taken from breast-cancer cells, exosomes have been proved to be involved breast cancer organ-specific metastasis. The underlying mechanism may be that exosomes expressed different cell-adhesion receptor proteins (called integrin) on their surface can target different organs and the specific organs recruited an abundance of ligand for the receptor. For instance, exosomal αvβ5 integrin directed to the liver, whereas α6β4 promoted homing to the lung [87] [88]. Thus, exosomal integrin profiles might predict organ-specific metastasis and act as a new biomarker in cancer diagnostics, and integrin inhibitors might curtail metastatic spread to specific organs.

## DUAL-EFFECT OF EXOSOMES IN TUMOR IMMUNE SYSTEM REGULATORY

Cancer cells escape the host immune system through manipulating their own immunogenicity and activation of immunosuppressive mediators [89]. Exosomes released biologically active molecules and posed modulatory effects on immune cells [90]. tumor released exosomes have been known for the diverse immunosuppressive effects in cancer progression [91]. One study by Wolfers *et al* reported exosome transfer tumor antigens from tumor cells to dendritic cells (DCs), and function in immune-interventions [92]. In most of these studies, exosomes delivered negative signals and posed suppressive effects on anti-tumour immune responses in breast cancer, as suppress specific T cell immunity and convert immune cells into pro-tumorigenic phenotypes [8]. Tumor-derived exosomes expressed functional CD39 and CD73, which resulted in de-phosphorylation of exogenous ATP and 5’AMP to procedure adenosine, and rising adenosine can attenuate T cell function [93]. DC-secreted exosomes (DCex) induced apoptosis by activation of caspase, activated natural killer cells and killed tumor cells [94]

Myeloid-derived suppressor cells (MDSCs) facilitated immunosuppressive functions and contributed to tumor progression [95]. Exosomes isolated from cancer-secreted exosomes which contained and promoted tumor growth [96]. Breast tumor-derived exosomes have also been found contributed to the growth of tumors by blocking IL-2-mediated activation of NK cells and their immune response to tumor cells [97]. Humor tumor-derived exosomes, as immune inhibitory, exosomal delivery of TGFβ1 to T cell or NK cell lead to down-regulation of NKG2D expression [98, 99]. Circulating Exosomes secreted by breast cancer contributed to NF-κB activation of macrophages and induced pro-inflammatory activity by upregulation of inflammatory cytokines [100].

Interestingly, studies have discovered that human breast milk contains exosomes with immune modulatory for the development of the infant's immune system [101]. Consistent with prior research conclusions, immune-related exosomal miRNAs from breast milk are resistant to general harsh conditions, and are essential for the development of infant immune system [102].

## EXOSOMES ACT AS MEDIATORS OF THERAPY RESISTANCE IN BREAST CANCER

**Figure 5:**
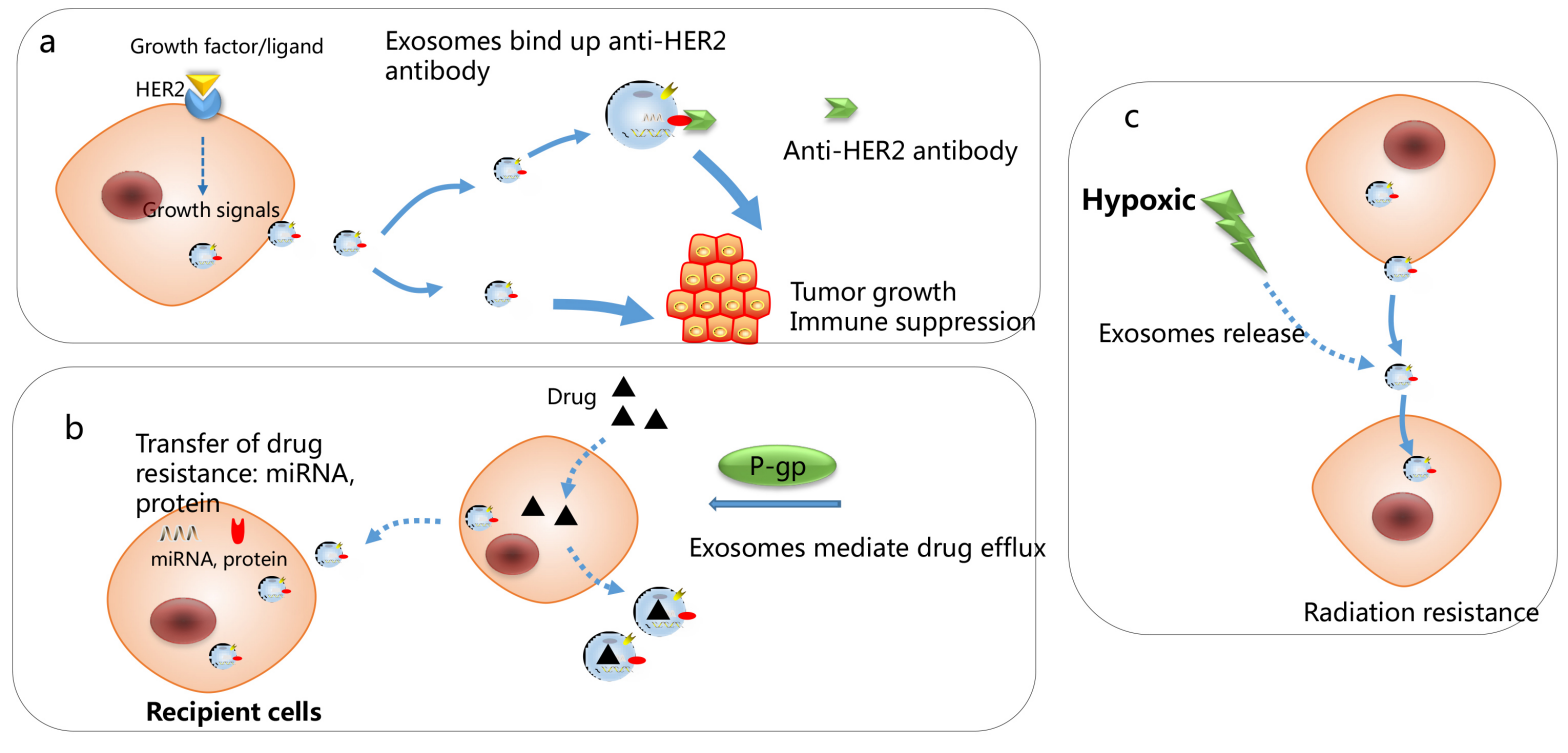
Mechanisms responsible for exosomes-associated drug resistance **a**. HER2 positive derived exosomes” could bind efficiently to Trastuzumab and inhibit the anti-proliferative effects of trastuzumab by preventing it from binding to tumor cells. **b**. Exosomes mediate drug efflux and transfer of drug resistance to recipient cells, through transfer of miRNAs and protein. **c**. Hypoxic-induced exosomes render cancer cells more resistant to irradiation.

Breast cancer therapy has received many restrictions due to therapy resistance, explanation of mechanisms responsible for drug resistance and seeking reliable indicators to predict the therapeutic effect is essential to achieve more effective and individualized chemotherapeutic treatment of breast cancer patients. Currently, some studies have shown that breast cancer exosome-mediated transfer of genetic information can induce therapy resistance and promote tumor progression, such as extruding hydrophilic drugs from cancer cells [103–105]. Studies also implied roles of exosomes in breast cancer radiotherapy resistance and cancer immunotherapy [106, 107]. (Figure 5)

**Figure 6:**
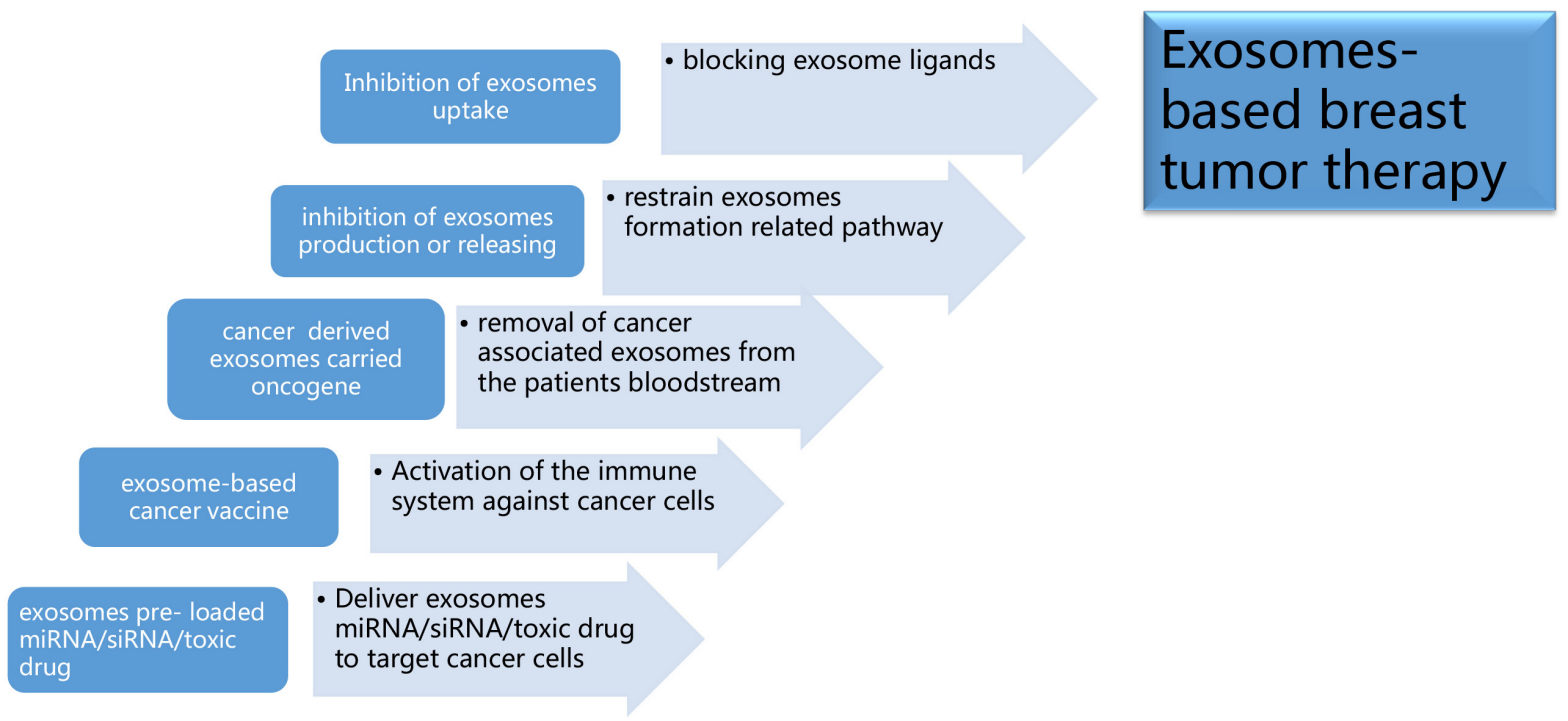
Exosomes-based breast tumor therapy **a**. Deliver exosomes containing miRNA, small interference RNA (siRNA), and/or anti-cancer drugs to target cancer cells; **b**. Activation of the immune system against cancer cells by implementing vaccines of exosomes containing proteins with unique or higher expression in tumor cell; **c**. To removal cancer or stromal derived exosomes containing oncogene related components from cancer patients’ circulation *via* molecule sponge or a hemodialysis-like process; **d**. To decrease oncogenic components containing exosomes production or releasing through targeting exosomes formation related pathway; **e**. Inhibition of exosomes uptake in recipient cells by blocking exosome ligands.

## CHEMOTHERAPY RESISTANCE

As breast cancer cells escape chemotherapy *via* various mechanisms, resistance to chemotherapy drugs remains a essential issue in the management of patients. As information transporters of intercellular communication, exosomes help cancer cells export chemotherapeutic drugs, which may facilitate the development of chemotherapy resistance. lead to chemo-resistance [5].

In breast cancer, drug-resistant breast cancer cells may spread resistance capacity and alter chemo-susceptibility in recipient sensitive cells *via* intercellular transfer of exosomal miRNAs (including miR-100, miR-222, and miR-30a) [107] [108]. The intercellular transport of P-glycoprotein (P-gp) was known to be involved in drug resistance *via* drug efflux across membranes [109, 110]. Exosomes mediated drug resistance *via* delivering P-gp from docetaxel-resistant cells to sensitive ones [111]. Jaiswal *et al* found that exosomes secreted by doxorubicin-resistant breast cancer cells contained abundant regulatory miRNAs (miR-451 and miR-326) and the latter conferred a more deleterious phenotype through “re-templating” recipient breast cancer cells’ transcriptional landscape to reflect the P-glycoprotein over-expressed character of donor cells [112]. Exosomes from doxorubicin-resistant breast cancer cells spread resistance capacity in recipient cells by delivering miRNAs, such as miR-100, miR-222, and miR-30a [113], and exosomal miRNAs can also transmit drug resistance to sensitive cells [114]. Exosomes derived from stromal fibroblasts transferred noncoding RNA to breast cancer cells and contribute to treatment resistance through expanding therapy-resistant tumor-initiating cells. [115].

## IRRADIATION THERAPY

Radiotherapy is a significant treatment method in breast cancer. Generally, oxygen is indispensable for radiation cytotoxic to cause DNA damage. Thus, breast cancer can be more resistant to radiation therapy under hypoxic microenvironment, and it has been shown that hypoxia leads to the increased production of exosomes [116] [117]. Besides, exosomes transferred from stromal to breast cancer cells stimulates antiviral signaling and derived radiation resistance in primary tumor [115]. Another study indicated that the proteome profiles of tumor-derived exosomes have been implicated in the indication of the oxygenation status of breast tumors [117].

## ENDOCRINE THERAPY AND TARGET THERAPY

Tamoxifen resistance remains a daunting challenge to the successful treatment of ER-positive breast cancer. In ER-positive breast cancer, exosomes secreted from tamoxifen resistant cells (MCF-7^TamR^) significantly promoted tamoxifen sensitive cells (MCF-7^wt^) proliferation and colony-forming ability in the presence of tamoxifen, and this effect was partly contributed to miRNAs (miR-221/222) enclosed in exosomes. miR-221/222 subsequently down-regulated its target genes of P27 and ERα protein levels, which caused increased tamoxifen resistance in recipient cells [118]

The clinical value of HER2-targeted therapies as trastuzumab and lapatinib is the most promising treatment for HER2 positive breast cancer patients targeted therapy. Despite this significant clinical benefit in HER2-positive patients, some patients exhibit intrinsic resistance to anti-HER2 therapy, and the underlying mechanism may be tumor cells utilizing alternative growth signaling or intracellular transduction molecules to bypass HER2 blockade [119, 120]. Tumor exosomes carrying antigens that are targets for anti-tumor antibodies, therefore competitively inhibit the drug activity and contribute to treatment failure. Consistent with these results, HER2 positive derived exosomes from BT-474 and SK-BR-3 cells interfered with the bioactivity of HER2-targeted therapeutic drugs by directing binding to Trastuzumab and prevented cancer cells from the anti-proliferative effects of trastuzumab. By contrast, no negative influence on the anti-proliferative activity of Lapatinib was observed [103, 121, 122]. The schematic in Figure 5 indicates the roles of cancer exosomes in modulating sensitivity to anti-HER2 therapy. Furthermore, HER2+ exosomes can be indicative of the stages of tumor development and HER2-related tumor aggressiveness. Exosomes isolated from HER2-positive advanced stage breast cancer patients’ serum presented higher-level binding ability to Trastuzumab compared to exosomes purified from the serum of those in early stage [107].

## EXOSOMES: FUTURE CHALLENGES

Exosomes have been proven to be important regulators in health and disease, especially in tumor biology. Tumor derived exosomes enclosing tumor-specific antigens and nucleic acids can be assessed non-invasive as potential diagnostic and predictive biomarker. Besides, exosomes could potentially be used to identify patients who are likely to develop metastatic disease, and the process of exosome production could yield new targets for cancer therapy.

## DIAGNOSIS AND PROGNOSIS MARKER

Cancer derived exosome release was accumulating in tumor cells, which could be isolated from serum, pleural effusions, urine, and ascites fluids of cancer patients [123]. Alterations in expression levels of certain exosomal biomarkers from body fluids of cancer patients propose a role for exosomes as an early marker for diagnosis and prognosis monitoring. In breast cancer patients, the levels of CEA and CA153 in circulating exosomes are linked to cancer progression [24] [123].

The biomarker potential for exosomes holds huge promise for diagnose and manage breast cancer. Circulating exosome-encapsulated microRNAs presented as an early ideal biomarker for breast cancer, and expression pattern of exosomal miRNAs is also correlated with tumor malignancy degree and prognosis [15, 125].. qRT-PCR and small RNA sequencing analysis of the microRNA content from plasma exosomes of patients with revealed that miR-21 and miR-1246 were detected significantly higher levels, as compared to those of healthy control [126]. Several miRNAs have been proved to be associated with breast tumor subtype and stage. Exosomal MiR-939 was found highly expressed in the basal-like tumor subtypes and associated with worse prognosis in triple-negative breast cancers [127]. One research has revealed that decreased several levels of exosomal miR-101 concentrations are associated with lymph-node positive cancer, and miR-373 levels were significantly upregulated in triple negative and ER and PR negative breast cancer patients [128]. By comparing tumor derived exosomes at baseline, pre-treatment and post-treatment, during follow up, and correlating with both clinical and pathologic response, we might also be able to predict therapeutic response and patient prognosis. Taken together, these reports suggested the roles of exosomes as promising biomarkers for the early diagnose of breast cancers and for evaluation of therapeutic effect and the patient's outcome.

## EXOSOMES-BASED THERAPY

Exosome involvement as carriers of cellular information in cancer progression is a promising strategy in the field of vectors for targeted drug delivery. And well established functional exosomes mimetics strongly increased the pharmaceutical acceptability of such drug delivery system [129]. *Via* drug delivery abilities for breast cancer target therapy, exosomes delivered chemotherapeutics such as doxorubicin to tumor tissue and suppressed cancer growth [130]. As natural carriers of miRNA, exosomes could be exploited as an RNA drug delivery system as cancer therapy. One study has examined that modified exosomes can be used to transfer nucleic acid drugs such as let-7a to epidermal growth factor receptor (EGFR)-positive breast cancer cells [131]. Recently, different groups have demonstrated that exosomes can be loaded on doxorubicin and with the aid of targeting moieties can efficiently release doxorubicin to different types of cancer cells [132]. In breast and ovarian cancer mouse models, exosomal doxorubicin significantly increased the therapeutic index of doxorubicin and limited the crossing of doxorubicin through the myocardial endothelial cells and avoiding accumulation of drug in the heart without affecting other organs. [133]. However, several essential issues need to be resolved to use exosomes more efficiently and widely. For instance, the drug delivery system requires that nucleic acid drugs can be efficiently transfected into exosomes, and the suitable host cells for exosome injection is an essential factor for future clinical applications.

Exosomes have been found to be involved in modulation of immune system, and the clinical use of exosomes showed promising results in cancer immunotherapy [134, 135]. Autologous dendritic cell-derived exosomes have been proved to be well tolerated in patients with advanced non-small cell lung cancer and metastatic melanoma [136, 137]. Therapeutic filtration of cancer exosomes from the circulation could alleviate exosome-mediated immune suppression and increase cancer immunotherapy efficacy [138]. Since tumor-derived exosomes contained various cytosolic and membranous tumor antigens, exosomes have been used as a nanoscale cancer vaccine and a promising candidate for cancer therapy [139].

## CONCLUSION

Exosomes can act as critical signal transduction facilitators between cancer cells and their recipient cells, *via* releasing a wide variety of biological molecules, such as miRNAs, proteins and their complexes, affecting the interaction of distant cancer cells in the tumor microenvironment and the breast cancer progress. In this review, we further elaborate the contribution of the cancer and stromal cells derived exosomes to regulating breast cancer progression. With the development of novel therapeutic strategies targeting or utilizing exosomes, it will lead to more effective prevention and intervention strategies in breast cancer therapy. However, it is critical to note that crucial contents of exosomes are still not fully elucidated, and there is still a long road to fully understand the role of exosomes in breast cancer progress. Exosome-based therapies, exosomes served as predictive and prognostic biomarkers still need to be firmly validated by further preclinical and multicenter clinical validation studies.
